# Re: Policy Versus Practice: Facilitators and Barriers of Chronic Care Integration in Dutch General Practice – a Survey Study

**DOI:** 10.5334/ijic.9873

**Published:** 2026-04-25

**Authors:** Daan P. Hurkmans, Eva de Bie

**Affiliations:** 1 Department of Public Health and Primary Care, Leiden University, Medical Centre, Leiden, The Netherlands; 2 Leiden University Medical Centre, Leiden, The Netherlands

**Keywords:** multimorbidity, integrated care, primary care, care groups, health policy, general practitioners

## Abstract

The aim of this Letter to Editor is to point out the impact of some of the article’s conclusions, while in our opinion these conclusions might not be accurate based on the detailed results. The authors report that 56% of health care providers in general practices do not feel capable of delivering integrated care. However, based on Table 2, this main conclusion appears inaccurate. Furthermore, it remains unclear which specific healthcare providers reported feeling incapable. We are concerned that the current interpretation may be misleading and we recommend correcting percentages and providing more detailed subgroup data in order to enhance the accuracy and clarity of the findings.

Dear Editor,

With great interest, we read the article by Toine E.P. Remers et al., titled “Policy Versus Practice: Facilitators and Barriers of Chronic Care Integration in Dutch General Practice – a Survey Study”, published in International Journal of Integrated Care, 2024, 2024; 24(4), 13, 1–11. The authors address an important topic, namely the integration of chronic care in Dutch general practice.

The authors report that 56% of health care providers in general practices do not feel capable of delivering integrated care. However, based on Table 2, this main conclusion appears inaccurate. Table 2 shows that 16% of respondents disagreed and only 1% strongly disagreed with the statement: “I feel capable of delivering integrated care to patients with complex care patterns”. The remaining 39%, who were included in the 56% cited in the article, neither agreed nor disagreed. This group cannot be automatically classified as feeling incapable; rather, their response suggests ambivalence or neutrality.

Furthermore, it remains unclear which specific healthcare providers reported feeling incapable—whether they were general practitioners, nurse practitioners, or healthcare providers in the ‘other’ category as outlined in Table 1. This additional detail would be helpful to interpret the results more accurately.

We are concerned that the current interpretation may be misleading, especially considering that the delivery of integrated chronic care is regarded as a core task of general practitioners in the Netherlands. Clarifying this aspect would strengthen the article’s conclusions and contribute to a more nuanced understanding of the challenges in chronic care integration.

We look forward to the authors’ response. We recommend correcting percentages and providing more detailed subgroup data in order to enhance the accuracy and clarity of the findings.

https://ijic.org/articles/10.5334/ijic.8443

